# Rethinking agency for genetic testing intention among Latinos: Determining predictors of intention for carrier screening and cancer predisposition testing

**DOI:** 10.1016/j.gim.2025.101455

**Published:** 2025-05-14

**Authors:** Daniel Chavez-Yenter, Kimberly A. Kaphingst

**Affiliations:** 1 Division of Hematology-Oncology, University of Pennsylvania, Philadelphia, PA; 2 Department of Medical Ethics and Health Policy, University of Pennsylvania, Philadelphia, PA; 3 Department of Communication, University of Utah, Salt Lake City, UT; 4 Huntsman Cancer Institute, University of Utah, Salt Lake City, UT

**Keywords:** Cancer predisposition testing, Carrier screening, Integrated behavioral model, Intention, Latino

## Abstract

**Purpose:**

Attitudes and clinical processes have been well studied in genetic testing contexts; however, studies tend to underutilize theoretical frameworks, especially among Latino groups. Behavioral intention is a consistent predictor of uptake of behavior and has many theoretical roots. Using the Integrated Behavioral Model, we modeled the relationships between attitudes, norms, and perceived agency with behavioral intention for both carrier screening (CS) and cancer predisposition testing (CPT) for a Latino-only cohort.

**Methods:**

Using structural equation modeling, we ran both measurement and structural models. An initial measurement model used confirmatory factor analysis for each latent variable (attitude, norms, and agency) and their indicators of the Integrated Behavioral Model. Factor loadings less than 0.50 were removed, and we subsequently ran a structural model with the outcome of intention to use CS and CPT.

**Results:**

Agency was the most consistent predictor for both testing types (b = 0.381, *P* < .01 – CS; b = 0.559, *P* < .01 – CPT), with attitudes being negatively associated with CS (b = −0.313, *P* < .05), whereas norms were positively associated (b = 0.350, *P* < .01).

**Conclusion:**

Many interventions among Latino groups tend to focus on education and attitude change. Our findings suggest that more effort should be made to address agency to empower patients for improved genetic testing access.

## Introduction

Rates of genetic testing for health purposes remain significantly lower for Latino populations than White populations.^1,2^ Specifically, rates of cancer predisposition testing (CPT),^1-3^ carrier screening (CS),^4,5^ prenatal testing,^6,7^ and newborn screening^8,9^ all remain low among Latino groups compared with White populations. As a result, Latino groups are not benefiting from potential hereditary cancer screenings, equal access of prenatal care, or preparation for any child born with a genetic condition.^2,8,9^ Although various explanations for these testing disparities have been identified (social determinants of health, clinical barriers, etc), most interventions designed for Latino populations have focused on educating Latino populations rather than targeting more upstream factors.^1,10,11^ Interventions in Arizona, Florida, and Texas have been successful in recruiting and improving genetic engagement and testing through the use of culturally concordant genetics providers and leveraging substantial resources to enhance the experiences for Latino groups.^12-15^ For others without these resources, however, other intervention approaches may be needed. Thus, this study sought to investigate the factors affecting Latino groups’ behavioral intention (using the Integrated Behavioral Model [IBM] as theoretical foundation) toward genetic testing across multiple testing contexts.

Gaining a strong sense of what factors could help improve behavioral intention to encourage genetic testing is often done without a theoretical foundation, and effective interventions may not be achievable without a theoretical foundation or conceptual framework guiding genetic testing service delivery. Although our study is not an intervention, previous research has found that much of the intervention research is atheoretical.^16^ As such, if other research groups are interested in implementing the same intervention, a theoretical foundation provides agreed-upon construct definitions and applications. Finally, meta-analyses would be unable to truly assess the impact of interventions across multiple studies; we argue that more theoretical foundations should be present in genetics research even if not a direct intervention. Although attitudes and clinical processes are well studied in genetic testing contexts,^10,17^ less focus has been placed on how individuals decide to test using a theoretical framework. The IBM describes the facilitators and barriers of behavioral intention to engage in the behavior, focusing on attitudes, as well as norms and self-efficacy beliefs that envelop factors that could facilitate or inhibit intention.^18^ However, the application of this theory in genetic testing contexts is limited.^16^

The IBM posits that any given behavior is likely to occur with a strong intention to perform the behavior, especially if one has the necessary skills and abilities to perform the behavior and there are no environmental (or other) constraints on performing the behavior.^18,19^ The IBM argues that these determinants (attitudes, norms, and agency) directly influence behavioral intention. Underlying these psychosocial determinants of intention are behavioral beliefs and outcomes evaluations (directly influencing attitudes), normative beliefs and motivation to comply (directly influencing norms), and control beliefs and perceived power (directly influencing agency). Behavioral beliefs and outcome evaluations reflect one’s understanding and beliefs whether a particular behavior will have a good, bad, or even uncertain outcome and have a notable influence on individuals’ attitudes. Specific referents significantly influence normative beliefs and motivation to comply. Referents refer to any family, friends, and/or community members an individual has. These referents are essential to consider because if one believes a behavior is deemed to be important (or not) and being performed by family, friends, and networks, the likelihood of motivation to comply in performing the behavior increases. Finally, perceived behavioral beliefs and perceived ability (self-efficacy) are crucial to an individual’s agency. Perceived behavioral control seeks to determine the perceived control, control belief, and perceived power a participant has around performing a behavior. Self-efficacy, on the other end, focuses on the perceived ability of the respondent to complete the behavior. The more one perceives that they are able and have the skills to perform a behavior, even in the face of barriers, that individual will have higher self-efficacy, perceived agency, and, subsequently, behavioral intention.

### Current applications of theory among Latino populations

Prior related research in the context of cancer predisposition testing and genome and exome sequencing has incorporated the IBM, Theory of Reasoned Action (TRA), or Theory of Planned Behavior (TPB) in their research design to explore potential associations.^20-25^ One such study was qualitative, using theory to generate hypotheses of how behavioral intention constructs influence parents’ attitudes on return of results of exome sequencing for their children.^23^ Other studies used either a mixed-method design or quantitative methodology to determine the relationships between IBM, Theory of Reasoned Action, and TPB constructs and behavioral intention toward genetic testing. This prior research found that attitudes were most strongly associated with behavioral intention for genetic testing.^20,21,26^ One study found that attitudes could be influenced by message exposure manipulations with negative or positive information.^21^ The other 2 of these studies applying TPB and IBM focused on 2 underserved communities, specifically Black^22^ and Latino^24^ communities. The study by Jones et al^22^ (2016), which was based in Michigan, found that Black populations underutilized CPT (specifically for hereditary breast and ovarian cancer), often because of lack of referral or recommendation from a health care provider. Sussner et al^24^ (2010) assessed the attitudes, behavioral beliefs, and perceived risks of genetic testing in New York City Latinas through interviews using the IBM constructs for question construction. Latina women in the study held positive beliefs and attitudes toward *BRCA* testing but also faced particular barriers. They often had lower awareness and knowledge of testing, more potential worry and distress about learning about cancer risks of family members, and perceived challenges with cost/insurance. Based on these findings, the authors developed a protocol for a culturally tailored narrative tool for *BRCA* counseling and testing.

### Current Study

Much of this previous research was published more than a decade ago,^24,25,27,28^ and substantial changes have occurred in genetic testing processing. Our study sought to inform intervention efforts and contribute broadly to the areas of genetic testing service delivery, communication, and health equity for Latino groups by finding a unique intersection that needs to be addressed and strengthened. Therefore, the goal of this study was to answer the following research questions:

RQ1: What IBM variables are associated with genetic testing intention for CS within a Latino-only cohort?

RQ2: What IBM variables are associated with genetic testing intention for CPT within a Latino-only cohort?

## Materials and Methods

### Participants and procedure

Using a cross-sectional English language survey online, we examined the associations of salient attitudes, facilitators, and barriers, drawing on IBM constructs to predict intentions to use genetic testing for CS and CPT. Participants recruited for this study were required to live in the United States, self-identify as Latino/a/e, be fluent in English, and be above the age of 18 years old. Qualtrics was used to distribute the survey to this fully Latino cohort. Qualtrics panels can collect representative samples and can be segmented into specific demographic groups.^29^ A total of 595 respondents were recruited by Qualtrics, stratified by gender. After validity checks (full completion, attention checks passed, agreeing to pay full attention to the survey, and respond honestly [rather than completing the full survey by just selecting 1 response]), 503 responses were used for analysis. The survey was conducted from October to November of 2023. Respondents received incentives from Qualtrics for their participation per panel guidelines (often $2-$5 dollars per survey completed). IRB approval was granted before the start of the survey.

### Survey development

The survey items to assess IBM constructs were developed based on wording from previous interviews when appropriate.^30,31^ Previous research using this approach has effectively measured attitudes, behaviors, and self-efficacy.^32-34^ In this study, the primary constructs of interest were attitude, perceived norms, and perceived agency. Subconstructs include affect, behavioral belief, evaluation, perceived value, injunctive norms, normative belief, motivation to comply, frequency, perceived control, perceived power, ability, and self-efficacy.

Survey questions used self-reported measures for the various constructs related to 2 genetic testing contexts: CS and CPT. These are the most common types of genetic testing that generally involve genetic counseling^35^ but differ in terms of populations, clinical processes, and reasons for testing. Because genetic tests have distinct motivations, processes, and unique facilitators and barriers, we elected to use 2 different contexts to determine if particular constructs are unique to each context. CS is typically done before a planned pregnancy to assess the likelihood of bearing a child with a genetic disorder. Although CS can also be done during pregnancy, pregnant persons may be offered prenatal testing (amniocentesis and chorionic villus sampling) instead.^35^ In contrast, CPT generally requires specialty care. Typically, a primary care provider (PCP) reviewing family history of cancer will refer an unaffected patient to germline testing to determine hereditary risk. If a patient has cancer, often oncologists will order somatic (tumor) testing, which can be paired with germline results if they meet testing criteria.^36^ Before the survey items, we provided instructions that included an overview definition of both CS and CPT for all participants and provided a hover-over feature of each genetics term in the survey to provide the definition if needed while completing the survey. The survey presented CS first followed by the survey items related to that specific genetic test followed by CPT. The majority of constructs measured participants’ own beliefs regarding genetic testing (Supplemental Survey).

### Variables

#### Dependent variable(s)

Our main outcome variable of interest was intention to use genetic testing (both CS and CPT). We asked 2 sets of questions to focus on both types of testing; therefore, we had 2 dependent variables. Intentions were proxied by asking, “In the next year, do you intend to use CS/CPT in a clinical setting?”

### Independent variables

#### Attitude

Attitudes were measured by 5 indicators within 2 subconstructs, experiential and instrumental attitude. These indicators—affect, experiential behavioral beliefs, evaluation, instrumental behavioral belief, and perceived value—were proxied for both types of genetic tests. All but 2 indicators were positively valanced; the 2 indicators that were not were reverse coded to match valance structure. Three scales were used to measure these items (Unpleasant to Pleasant, Unlikely to Likely, and Bad to Good) on a Likert-like range, 1 to 5. Initial reliability measures were calculated, with Cronbach alpha values of.638 for CS and.541 for CPT. Structural Equational Modeling (SEM; discussed more in depth below) runs regression and confirmatory factor analysis simultaneously and was used to identify poor performing indicators of each independent variable. As such, 2 indicators for attitude were dropped as a result from the confirmatory factor analysis (CFA).

#### Perceived norms (family and friends)

Perceived norms were measured by 5 indicators within 2 subconstructs, subjective norms and descriptive norms. Subjective norms gauged respondents’ general approval or disapproval of genetic testing and how they perceived the general population and their family and friends’ networks approval or disapproval of genetic testing (injunctive norm, normative belief, and motivation to comply). Descriptive norms asked respondents to reflect on how popular and prevalent genetic testing is in its use (frequency and normative belief). One scale was used for these items using a Disagree to Agree Likert-like range (1 to 5). Initial Cronbach alpha values were 0.738 for CS and 0.749 for CPT. One indicator was dropped as a result from the CFA of the SEM.

#### Perceived norms (primary care provider)

In our prior work,^30^ a key facilitator for many respondents who tested was their provider. To test the effect of these norms in the survey, we assessed subjective norms constructs (3 indicators—injunctive norm, normative belief, and motivation to comply) for a PCP referent. For example, an item was “My primary care doctor approves using carrier screening/CPT.” One scale was used for these items using a Disagree to Agree Likert-like range (1 to 5). Initial Cronbach alpha values were0.771 for CS and 0.736 for CPT.

#### Perceived norms (other doctors/specialists)

Our prior work showed that there were notable differences in knowledge and facilitation of testing from oncologists, surgeons, or other specialists compared with PCPs. As such, we added subjective norms constructs with the same 3 indicators, injunctive norm, normative belief, and motivation to comply, changing the referent group from PCPs to other doctors and specialists. For example, an item was “My other healthcare providers (oncologist, OB-GYN, etc) approve using carrier screening/CPT.” One scale was used for these items using a Disagree to Agree Likert-like range (1 to 5). Initial Cronbach alpha values were 0.759 for CS and 0.780 for CPT.

#### Perceived agency

Perceived agency asked respondents to report their own beliefs in their ability to get a genetic test within 2 subconstructs, perceived behavioral control and self-efficacy, comprising 5 indicators. Perceived behavioral control had 3 items to proxy the construct, perceived control, control belief, and perceived power. Self-efficacy focuses on the ability of the respondent and uses 2 items to proxy the construct, ability and self-efficacy belief. Three scales were used for these items (Difficult to Easy, Not Confident at all to Completely Confident, and Disagree to Agree) on a Likert-like range, 1 to 5. Initial Cronbach alpha values were 0.804 for CS and 0.814 for CPT. One indicator was dropped as a result from the CFA of the SEM.

#### Demographic and clinical covariates

Other variables of interest included both demographic and clinical features of the respondents. Demographics collected included gender, age, education level, subethnicity (country of origin/subethnic identification), race, bilingual status, preferred language, marital status, and income. Clinical variables collected included insurance, having a regular PCP, having biological children, previous cancer diagnosis, and previous genetic testing.

## Data analysis

First, we ran descriptive analysis on demographic characteristics, clinical covariates, and the dependent variables of intention to use CS and CPT. Next, SEM was used in 2 ways. SEM has the ability to run both CFA and a regression analysis simultaneously and thus was an ideal analytical approach.^37^ First, we used a measurement model to test the strength of variable items’ relationships with our IBM constructs using CFA (Supplemental Figures 1-4). Next, after identifying factor loadings less than 0.50, we dropped them from the model for better model fit (Table 1).

Second, we used a structural model to run both factor analysis and regression simultaneously with intention to use CS and CPT with IBM constructs, including demographic and clinical covariates. Regression models presented in the results show standardized coefficients. In the structural models, we report the χ^2^ result (*P* < .05 good fit), comparative fit index (CFI, acceptable fit >0.90, good fit >0.95), and root mean square error of approximation (RMESA, <0.05 good fit) to show model fit.^37^ Akaike Information Criteria (AIC) and Bayesian Information Criteria (BIC) are also reported as is standard practice in SEM reporting.^37^ Analysis was completed using R (R Core Team, 2024) and, specifically, the packages “laavan” and “psych.”^38,39^ Results were considered statistically significant if they reported values *P* < .05, and were marginally significant if *P* < .10.

## Results

### Descriptive

A total of 503 Latino individuals completed the survey via Qualtrics and were included in the analyses. Both demographic characteristics and clinical information are presented in Table 2. Respondents reported a mean age of 35.9 years old (range of 18–82 years old). The majority were female (52%), had a high school/GED or college degree or higher (71%), considered themselves bilingual (73%), were insured (86%), had not previously had genetic testing (93%), were of Mexican origin (50%), and White (53%). In relation to the primary outcome of interest, intention to test, respondents were fairly equally distributed between unlikely/somewhat unlikely, unsure, and likely/somewhat likely for both CS and CPT, with approximately a third of respondents in each of these categories (Table 3).

### Structural equation modeling

#### Measurement models

To determine if any indicators for the latent variables were poor proxies, we elected to run a CFA using SEM. The first model that was developed was the measurement model (running a CFA alone). First for *CS*, we ran a CFA of our latent/exogenous variables (IBM constructs) with their indicators and were able to calculate their factor loadings (Table 1). Factor loadings add to correlations by also describing the variability among indicators and their relation to a given factor. Experiential behavioral belief and instrumental behavioral belief both had poor factor loading for attitude (0.189 and 0.046, respectively). Additionally, other indicators that had poor fit (<0.50) included motivation to comply to norms for family and friends and perceived power within agency. With norms for PCPs and other doctors/specialists, only motivation to comply had a poor factor loading (<0.50).

For *CPT*, the same measures had poor factor loadings, with experiential behavioral belief and instrumental behavioral belief having negative factor loadings with attitude, as well as motivation to comply for family and friends and perceived power having factor loadings less than 0.50. Norms for PCPs and other doctors/specialists performed well with factor loadings.

#### Revised measurement model

As a result of the CFA for CS, we elected to drop the indicator variables that had factor loadings less than 0.50 (with the exception of motivation to comply for PCP norms). Thus, attitude, norms for family and friends, and agency were revised to drop items with poor factor loadings. Norms for PCPs and other doctors/specialists factor loadings largely did not change with the exclusion of attitude, norms, and agency indicators (Table 1). Although motivation to comply did have a poor factor loading, it was not excluded from the model to have a minimum of 3 indicators for each latent variable. Because the value0.487 approached the 0.50 threshold for inclusion, we opted to keep this indicator in the model.

For CPT, based on the CFA results, we excluded the same indicators as CS, which resulted in the acceptable factor loadings for each indicator for constructs. For norms for PCPs and other doctors/specialists, no significant changes were noted, and all factor loadings were ranged from acceptable to good (>0.50-0.90).

#### Structural model

**Figure 1:**
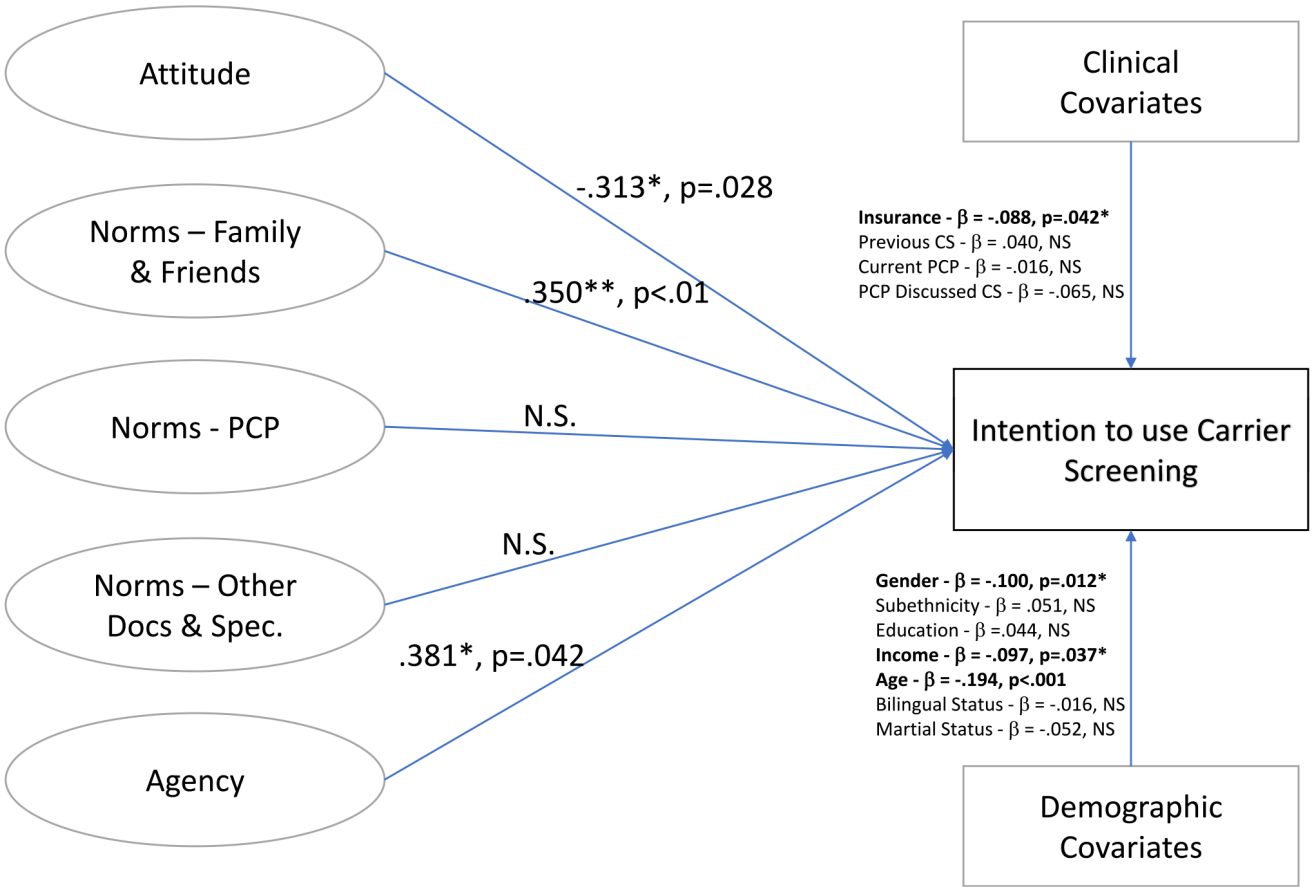
Structural model of latent variables on carrier screening intention (CFI = 0.902; AIC = 20,483.244; BIC = 20,740.582; RMESA = 0.051). AIC, Akaike Information Criteria; BIC, Bayesian Information Criteria; CS, carrier screening; CFI, comparative fit index; NS, non-significant; PCP, primary care provider; RMESA, root mean square error of approximation.

Next, a structural model was run to show the relationships between the endogenous/dependent variable of intention to use CS and CPT with the revised exogenous/latent variables, including their factor analysis (including demographic and clinical covariates). We therefore report 2 models. First, we present the structural model with standardized regression estimates for CS intention (Figure 1). Attitude (b = −0.313, *P* = .028), norms of family and friends (b = 0.350, *P* < .01), and agency (b = 0.381, *P* = .042) were significant predictors. Model fit parameters were all within acceptable ranges (χ^2^ = 675.3, df = 307, *P* < .001, CFI = 0.902, AIC = 20483.244, BIC = 20740.582, RMESA = 0.051). Significant demographic indicators included gender (b = −0.100, *P* = .012), income (b = −0.094, *P* = .037), and age (b = −0.194, *P* < .001). The only clinical covariate that was significantly associated with intention to use CS was insurance status1 (b = −0.088, *P* = 0.42). Another structural model was run excluding those who had previously tested (*n* = 35), but it did not significantly change the results of effect sizes and impact on intention for CS testing (Supplemental Figure 5).

**Figure 2:**
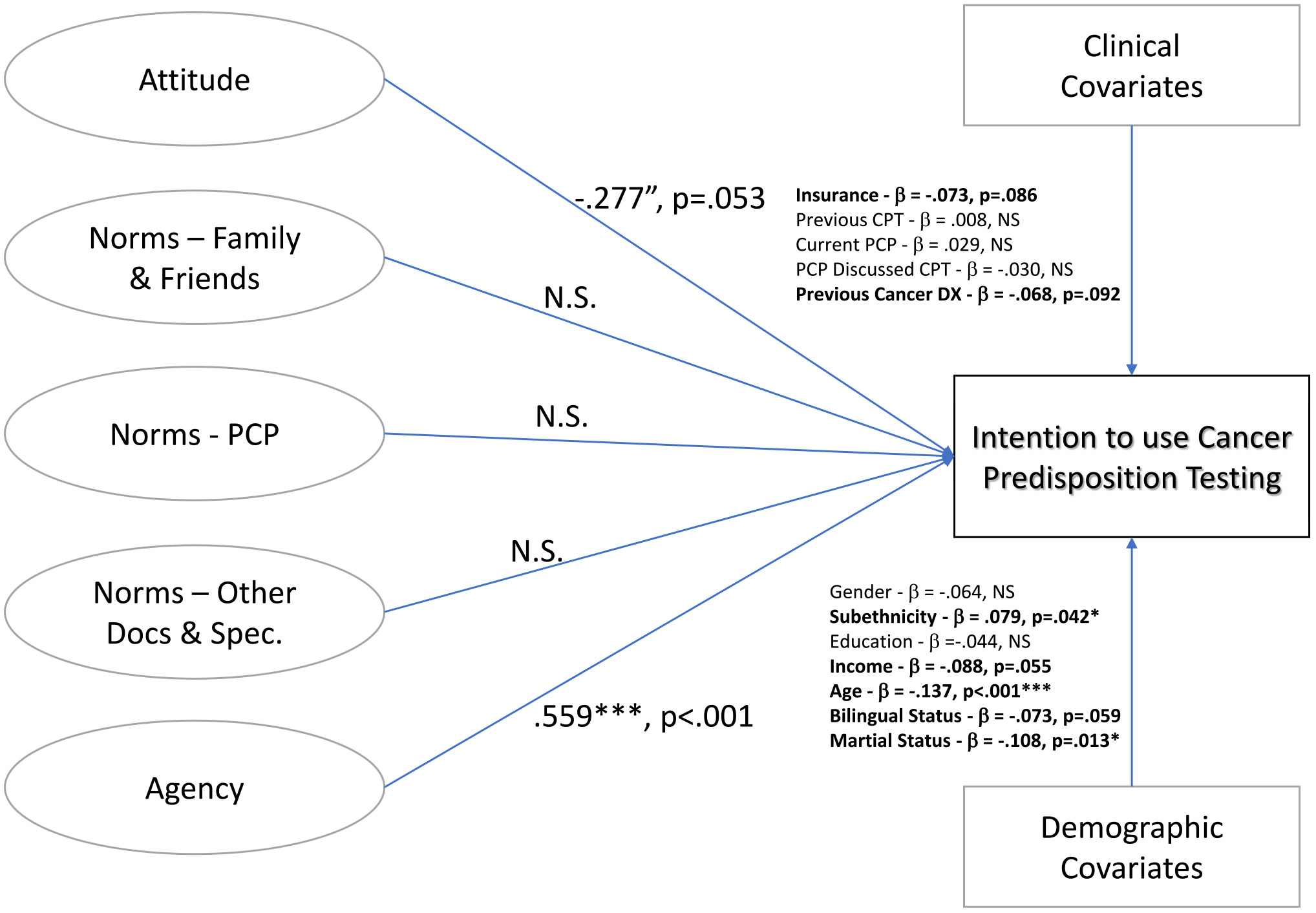
Structural Model of Latent Variables on Cancer Predisposition Testing Intention (CFI = 0.924; AIC = 19,843.552; BIC = 20,100.091; RMESA = 0.047). AIC, Akaike Information Criteria; BIC, Bayesian Information Criteria; CFI, comparative fit index; CPT, cancer predisposition testing; DX, diagnosis; NS, non-significant; PCP, primary care provider; RMESA, root mean square error of approximation.

Second, we present here the structural model for *CPT* with the revised latent variables based upon the measurement model. Listed are the standardized regression estimates between latent variables, demographic covariates, and clinical covariates on intention to use CPT (Figure 2). Attitude had a marginally significant association, with a negative effect on intention for CPT (b = −0.277, *P* = .053), whereas norms for family and friends, PCPs, and other doctors/specialists were all nonsignificant in the model. But agency was significantly associated with intention for CPT (b = 0.559, *P* < .001). Model fit for this structural model was good (χ^2^ = 653.7, df= 325, *P* < .001, CFI = 0.924, AIC = 19843.552, BIC = 20100.091, RMESA = 0.047). Significant demographic predictors were subethnicity (b = 0.079, *P* = .042), age (b = −0.137, *P* < .001), and marital status (b = −0.108, *P* = .013). Predictors that were marginally significant included income (b = −0.088, *P* = .055), bilingual status (b = −0.073, *P* = .059), insurance status (b = −0.073, *P* = .086), and previous cancer diagnosis (b = −0.068, *P* = .092). Another structural model was ran that removed the 35 participants who previously tested and nullified IBM construct effects on intention to test (Supplemental Figure 6).

## Discussion

Based on our findings, IBM constructs appear to be important predictors of genetic testing intentions for Latino populations and yet may have distinct relationships for different types of testing. For CS, attitudes, norms for family and friends, and agency were all significant predictors for intention to test, suggesting that those with more positive attitudes, normative influence from family and friends, and self-belief they can seek testing will do so. More attention may be needed to changing individual attitudes toward CS to promote genetic services engagement. Current efforts to improve knowledge and awareness of genetic testing for Latino populations^12,15^ would be supported by our results. However, more efforts could be designed to leverage norms for family and friends and agency. For example, interventions designed to encourage family members or friends to discuss benefits of testing related to family planning or being able to prevent certain conditions may be a beneficial approach, particularly because norms from family and friends are influential for Latino populations.^11,40-42^ This type of approach may also positively affect intention through norms of family and friends. Interventions could also use family networks and friends to promote testing.^41^ Despite our nonsignificant finding of normative influence of PCPs or other doctors/specialists on intention to testing here, other research has found a desire from providers to direct the process of testing.^30^ However, this normative influence has not been rigorously studied with the Latino population; therefore, our null finding may reflect the reality of PCP influence on testing intention for this population. Addressing agency likely would need more clinical-level intervention, such as navigation interventions to address barriers (designating a clinical staff support member for education,^43^ automation,^44^ etc).

For CPT, agency was the strongest predictor for testing intention, although not for those who had previously tested. We found that those who had tested previously had notably higher self-reports of agency than the rest of the population, thus potentially biasing results. However, other IBM constructs did not significantly differ. Nevertheless, because CPT often requires specialty care and a referral process, it highlights that more efforts are likely needed at the clinical level to improve individual perceptions of agency. For example, flyers could be placed in waiting rooms or examination rooms, prompting patients to ask about their provider about genetic testing, or a designated clinical staff member could prescreen patients to determine if they are eligible for genetic testing to take the workload off the provider. If an intervention approach can empower patients, our results suggest that they are more likely to follow through with testing uptake. Interventions could be designed to address all 3 constructs (attitudes, norms for family and friends, and agency); however, it appears that norms for family and friends and agency may be more fruitful areas for intervention, diverging from much of the prior literature that has focused on attitudes and knowledge.^17^

Finally, although the effects had a small influence, we did find significant associations between demographic and clinical covariates and intention to test for both CS and CPT. Gender, age, income, marital status, and subethnicity were significant predictors in both models, consistent with prior literature.^2,40,45^ In other words, women, older individuals, higher income, those who are married, and certain Latino ethnic groups (often Puerto Rican or Cuban) were more likely to have a higher intention to test.^1,2,46,47^ Often these demographics are tied to class status, highlighting the importance of social determinants’ influence of intention to test (and uptake). Insurance is often cited as a barrier to testing^40,41,48,49^ and is likely critical to improving interventions. As an external environmental factor, even if an individual has positive attitudes, normative influence, and high agency, it may not matter if insurance remains a barrier to testing. Thus, insurance needs full consideration when developing interventions for Latino groups.

Although, to our knowledge, only 1 other study previously analyzed similar behavioral constructs using the TPB and SEM methods with intention and testing uptake,^25^ our results are an important contribution, finding similar results with a Latino-only cohort. In the Wade et al^25^ study, positive attitudes toward genetic testing were the strongest predictor of testing intention among a predominately White cohort (although not exclusive to that population). Particular demographic characteristics were also associated with testing uptake; older, White, and college-educated participants were more interested in testing than others in the study, suggesting that older, higher educated, or White individuals would be more likely to use genetic testing, mirroring our results. Although norms and agency part of the IBM were not assessed, Wade et al^25^ found within the TPB that perceived severity, worry, and response efficacy all had influences, and all contributed to attitudes that could be addressed when developing interventions. Although our results found similar trends around attitude, our contribution went further to assess norms for family and friends, PCPs, other doctors/specialists, and agency with intention to test.

### Clinical implications

Although agency is largely focused on the individual within the IBM theoretical foundation, many factors that can affect agency are external. In other words, to improve agency, one needs to empower patients to feel that they can seek out genetic testing to improve testing intention. Interventions may therefore need to address more institutional- or clinical-level factors, such as access to testing, improving referral processes, and offering an opportunity to learn more about testing, and ask questions to address uncertainties that may reduce agency.^15,43,50^ Delivery of genetic services involves a multitude of structural, institutional, provider, and patient factors. Much of the recent research on the Latino experience with genetic testing has focused on patient challenges (our study included), and critical analysis is needed of how health care institutions, genetics providers, and PCPs affect intention to test.^41^

Although intention often is strongly correlated with behavior uptake,^32,51^ there has been a notable amount of research that has more critically analyzed the intention-behavior relation.^52^ Although Latino groups may intend to use genetic testing, when it comes to actually seeking out testing, a lack of confidence in being able to access testing may prevent them from actual uptake. This suggests the importance of communication from health care systems and providers about concrete steps that participants can take to access genetic testing to improve individual agency. Increased telegenetics efforts or online delivery of care could help PCPs order and provide testing for all patients,^53^ especially for those in rural and underserved communities. Improving agency may benefit both patients and providers. Patients can be empowered to learn about their potential genetic predisposition to certain cancers or genetic conditions while advocating for their own testing. Providers, on the other hand, can be empowered to apply these results to provide more personalized medical care for their patients with better outcomes for screenings, cascade testing, and more, with improved genetic service delivery practices.

### Conclusion

Our study is not without limitations. First, although the SEM model seemed to explain a substantial amount of the variance in testing intention, there are likely other factors that influence intention. Previous testing appears to affect intention for other types of testing. Although excluding these participants who had previously tested in an alternate model did not affect results significantly for CS, all IBM constructs for CPT were nullified. Descriptive results did not show significant differences on IBM constructs and clinical and demographic covariates between testers and non-testers, leading us to include them in the model presented here. Therefore, IBM constructs should be examined further in studies that include larger samples of both individuals who have previously tested and those who have not. Second, attitude in both models appeared to be a poor latent variable, possibly as a result of item wording. More research is needed on how attitude is proxied, especially within the Latino community because these items may not capture attitude for Latino-specific populations. Third, Qualtrics panels may not be generalizable to the whole population because those who participate may be more technologically literate or move through surveys quickly to simply obtain the financial incentive or could even be bots created to farm responses. We attempted to address this challenge with attention checks and validity checks on survey completions. Fourth, the presentation of CS and CPT in order may have affected participants responses, being unable to differentiate between the 2 genetic tests and their implications. Fifth, oversaturation of our model is a potential concern. However, when models were ran with only significant covariates, model fit parameters actually worsened, whereas the main IBM effects were largely unchanged. We believe that the results we present here capture the accurate interpretation of the results. Finally, we only surveyed English-fluent Latino individuals using an English language survey. Older generations report using technology at lower rates compared with their younger counterparts and tend to prefer the use of Spanish to English.^54,55^ Future research should include Spanish-speaking Latino populations to determine if the same constructs are predictors for testing intention. Based on our results, norms for family and friends and agency are prime for intervention. These types of interventions should work with genetic providers to ensure implementation of programs that can facilitate familial communication, as well as improving access to genetic testing. Although genetic testing should not be considered the right option for all patients, it is important to ensure equity among its availability for all patients.

## Supplementary Material

1

2

The online version of this article (https://doi.org/10.1016/j.gim.2025.101455) contains supplemental material, which is available to authorized users.

## Figures and Tables

**Table 1:** Confirmatory factor loadings for carrier screening and cancer predisposition testing of measurement models

Construct	CS Model Factor Loading	CPT Model Factor Loading	CS Model Revised Factor Loading	CPT Model Revised Factor Loading
Attitude Affect	0.654	0.622	0.657	0.616
Exp. Behavioral Belief	0.189	−.052	Dropped	Dropped
Evaluation	0.801	0.752	0.792	0.763
Inst. Behavioral Belief	0.046	−0.083	Dropped	Dropped
Perceived Value Norms	0.759	0.734	0.746	0.730
Inj. Norms	0.694	0.710	0.708	0.706
Norm Belief	0.678	0.723	0.719	0.729
Motivation to Comply	0.454	0.479	Dropped	Dropped
Frequency	0.597	0.633	0.545	0.610
Desc. Normative Belief	0.578	0.602	0.537	0.582
Norms (PCP) Inj. Norms	0.758	0.807	0.759	0.809
Norm Belief	0.764	0.771	0.756	0.772
Motivation to Comply Norms (ODS)	0.490	0.653	0.487	0.650
Inj. Norms	0.661	0.783	0.785	0.784
Norm Belief	0.723	0.789	0.727	0.791
Motivation to Comply Agency	0.583	0.716	0.586	0.712
Perceived Control	0.675	0.699	0.759	0.691
Control Belief	0.709	0.667	0.718	0.749
Perceived Power	0.324	0.484	Dropped	Dropped
Ability	0.758	0.725	0.718	0.732
Self-Efficacy Belief	0.709	0.725	0.756	0.749

CS, carrier screening; CPT, cancer predisposition testing; ODS, other doctors/specialists; PCP, primary care provider.

**Table 2:** Demographic and clinical covariates of survey respondents

Variable	*n* (%)
Demographics	*N* = 503
Gender	
Female	259 (51.9%)
Male	241 (47.9%)
Nonbinary	3 (0.59%)
Education	
Elementary	4 (0.80%)
Junior high	22 (4.39%)
High school/GED	151 (30.1%)
Some college	119 (23.8%)
Associate degree	66 (13.2%)
College degree	96 (19.2%)
Graduate degree	43 (8.6%)
Subethnicity*	
Mexican	283 (50.1%)
Puerto Rican	84 (14.9%)
Cuban	36 (6.4%)
Dominican	21 (3.7%)
Other	21 (3.7%)
Salvadoran	19 (3.4%)
Costa Rican	12 (2.1%)
Colombian	12 (2.1%)
Venezuelan	10 (1.8%)
Guatemalan	9 (1.6%)
Argentine	9 (1.4%)
Ecuadorian	7 (1.2%)
Peruvian	7 (1.2%)
Honduran	6 (1.1%)
Nicaraguan	6 (1.1%)
Panamanian	5 (1.0%)
Paraguayan	5 (1.0%)
Chilean	4 (0.71%)
Bolivarian	2 (0.40%)
Race^a^	
White	285 (53.3%)
Afro-Latino/American	95 (17.8%)
Asian/Pacific Islander	22 (4.1%)
Native American	36 (6.7%)
Other	97 (18.1%)
Bilingual status	
Yes	366 (72.9%)
No	136 (27.1%)
Marital status	
Married	149 (30.0%)
Living as married	54 (11.8%)
Widowed	15 (3.0%)
Divorced	32 (6.4%)
Separated	10 (2.0%)
Never married	241 (48.1%)
Household Income	
Less than $25,000	108 (21.5%)
$25,000-$49,999	145 (29.0%)
$50,000-$74,999	118 (23.5%)
$75,000-$99,999	80 (15.9%)
$100,000 or higher	51 (10.2%)
Age (mean, SD)	35.9 (13.9)
Clinical	
Have insurance	
Yes	430 (86.5%)
No	67 (13.5%)
Have a PCP	
Yes	370 (74.0%)
No	112 (22.4%)
Not sure	18 (3.6%)
Have a biological child	
Yes	279 (55.5%)
No	224 (44.5%)
Previous cancer diagnosis	
Yes	34 (6.8%)
No	467 (93.2%)
Previous testing in clinic	
Yes	35 (7.0%)
No	462 (93.0%)

PCP, primary care provider.

a Respondents could have selected more than 1 response.

**Table 3:** Interest in testing for carrier screening and cancer predisposing testing

Interest in Testing	CS, *n* (%)	CPT, *n* (%)
Unlikely	97 (19.4%)	77 (15.4%)
Somewhat Unlikely	53 (10.9%)	41 (8.2%)
Unsure	174 (34.9%)	187 (37.5%)
Somewhat Likely	110 (22.0%)	115 (23.1%)
Likely	66 (13.2%)	79 (15.8%)

CS, carrier screening; CPT, cancer predisposition testing.

## Data Availability

Data are available from the authors upon request. Contact Dr Daniel Chavez-Yenter for data inquiries.
