# Supplementary material for: Rethinking agency for genetic testing intention among Latinos: Determining predictors of intention for carrier screening and cancer predisposition testing
Source: Genet Med. Author manuscript; Available in PMC 2026 Apr 20. (PMC13094735; doi:10.1016/j.gim.2025.101455)
Supplement: 1 [file NIHMS2162006-supplement-1.docx]

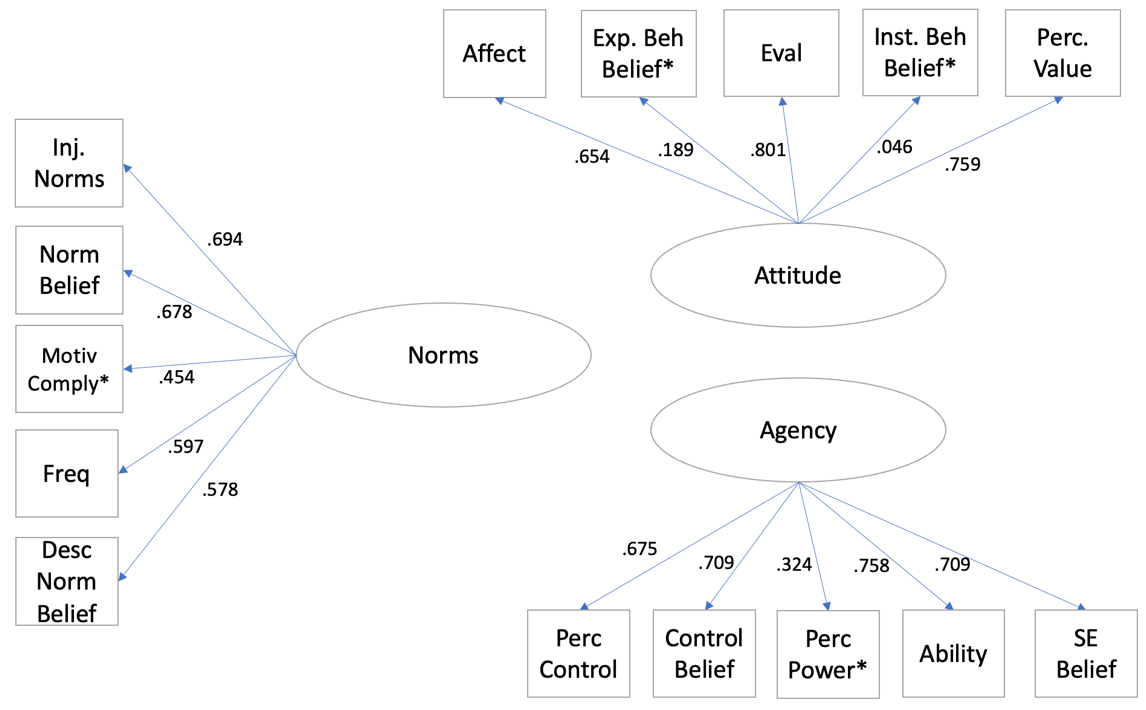


Supplemental Figure 1. Measurement Model of IBM Constructs With Factor Loadings for Carrier Screening


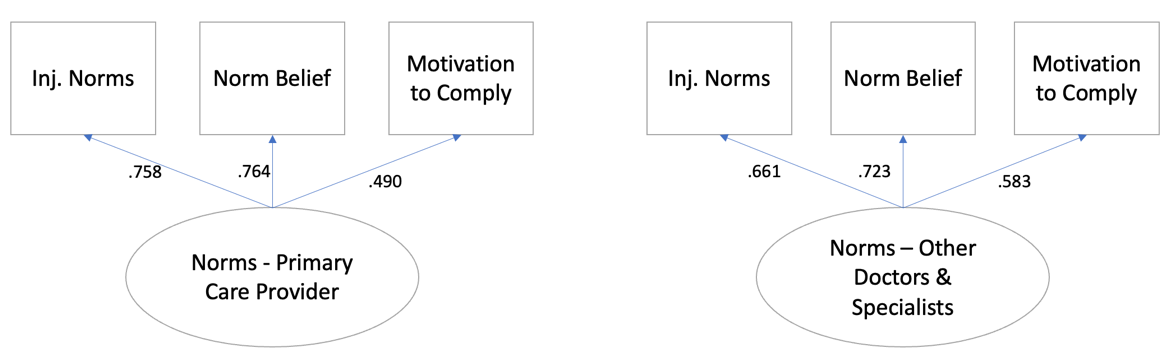


Supplemental Figure 2. Factor Loadings for Norms for PCPs and Other Doctors/Specialists for Carrier Screening


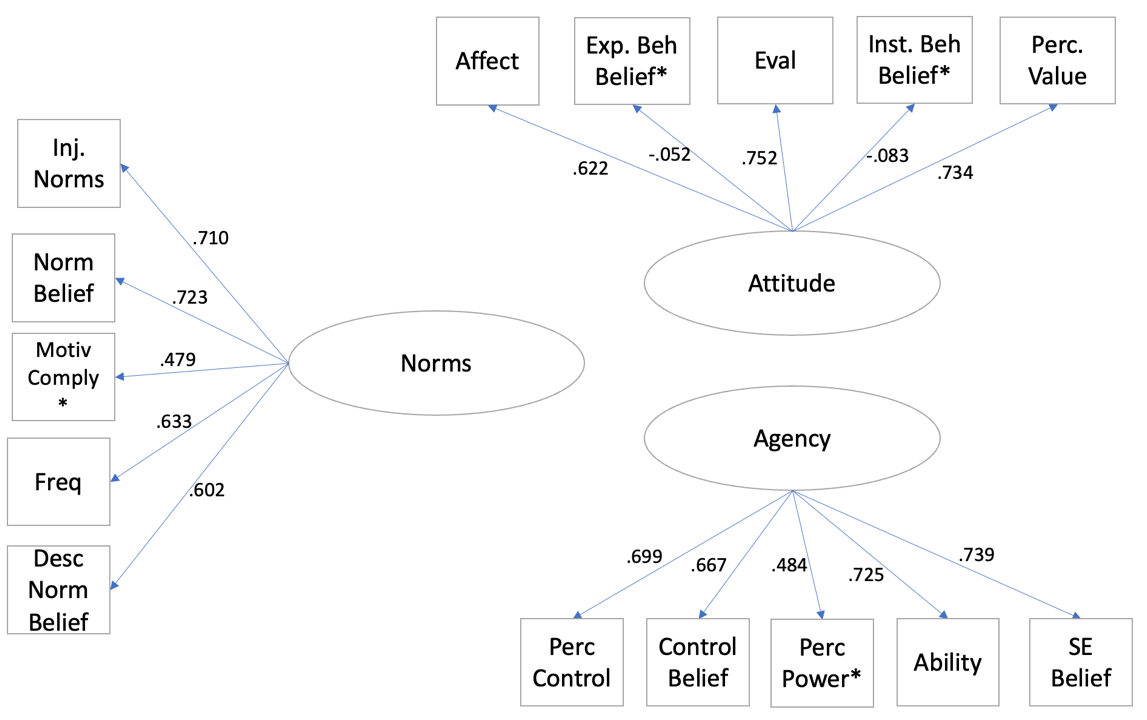


Supplemental Figure 3. Measurement Model of IBM Constructs With Factor Loadings for Cancer Predisposition Testing


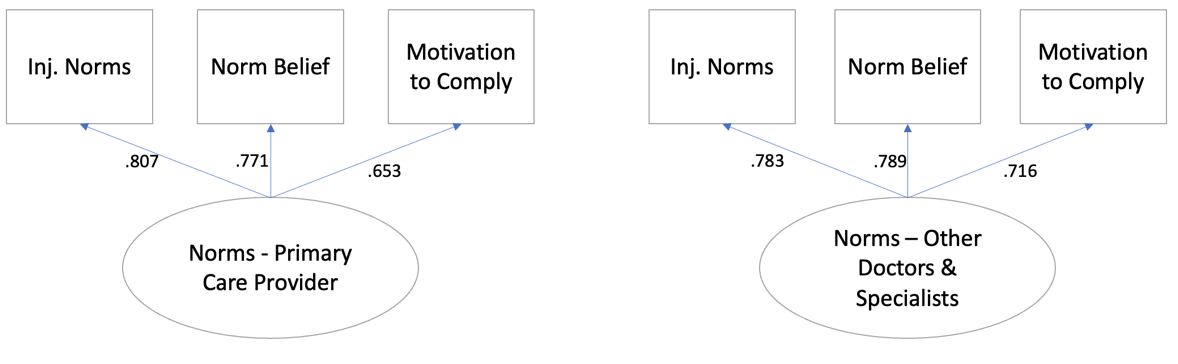


Supplemental Figure 4. Factor Loadings for Norms for PCPs and Other Doctors/Specialists for Cancer Predisposition Testing


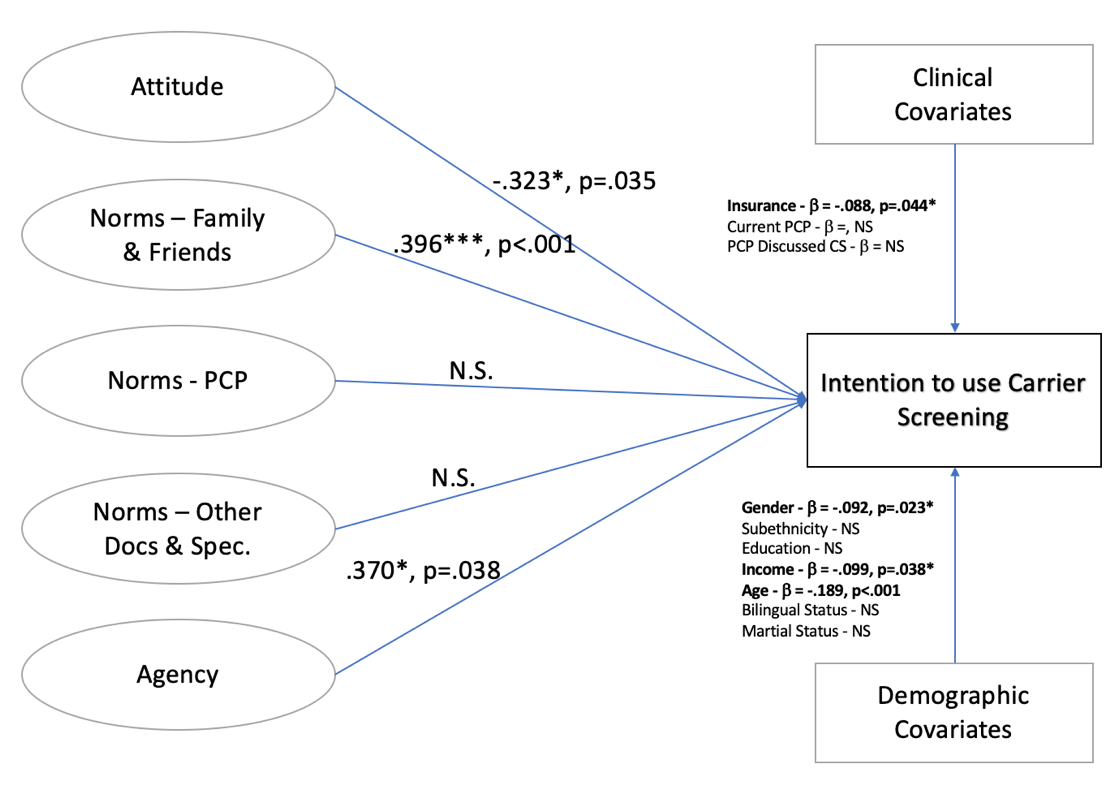


Chi-Square=634.04, df=290, p<.001, CFI = .903, AIC=19225.86, BIC=19475.14, RMESA=.052

Supplemental Figure 5 - Revised Carrier Screening Structural Model Excluding Individuals who have Previously Tested


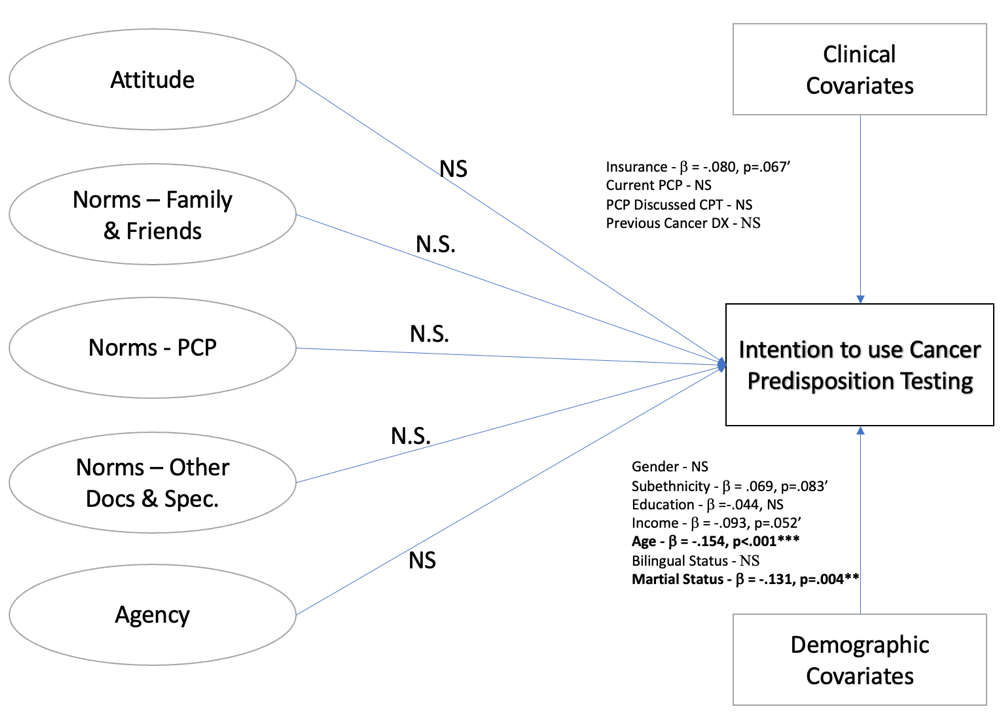


Chi-square=633.99, df=308, p<.001, CFI=.920, AIC=18458.56, BIC=18706.73, RMESA=.049

Supplemental Figure 6 – Revised Cancer Predisposition Testing Structural Model Excluding Individuals who have Previously Tested
