# Supplementary material for: Rethinking agency for genetic testing intention among Latinos: Determining predictors of intention for carrier screening and cancer predisposition testing
Source: Genet Med. Author manuscript; Available in PMC 2026 Apr 20. (PMC13094735; doi:10.1016/j.gim.2025.101455)
Supplement: 2 [file NIHMS2162006-supplement-2.docx]

**Aim 2 – Survey Questions regarding Genetic Testing**

**Demographics**

1. **How old are you?**

___________________

1. **What is the highest level of school that you have completed?**

- Elementary school
- Junior high or some High school
- High School or GED
- Some college or Associate degree
- College degree
- Graduate degree

1. **Do you consider yourself: Please select all that apply.**

- Mexican
- Guatemalan
- Honduran
- Nicaraguan
- Salvadoran
- Costa Rican
- Panamanian
- Colombian
- Venezuelan
- Ecuadorian
- Peruvian
- Bolivian
- Paraguayan
- Chilean
- Argentine
- Uruguayan
- Dominican
- Cuban
- Puerto Rican
- Other - Please Specify: _____________

1. **What is your race? Select all that apply.**

- White/Caucasian
- African-Latino & American/Black
- Asian/Pacific Islander
- Native American/Alaska Native
- Other - Please specify: __________________________________

1. **Do you consider yourself bilingual in Spanish?**

- Yes
- No

1. **What language do you typically use the most?**

- English
- Spanish
- Both English and Spanish Equally
- Other: Please specify: _______________

1. **Do your parents or grandparents have any Ashkenazi (Eastern European) Jewish ancestry?**

- Yes
- No
- Maybe
- Don’t Know

1. **What is your marital status?**

- Married
- Living as married
- Widowed
- Divorced
- Separated
- Never been married

1. **What is your zip code?**

__________________________________

1. **Think about your household's total income. About how much did your household receive in the last year?**

- Less than $25,000
- $25,000 - $49,999
- $50,000 - $74,999
- $75,000 - $99,999
- $100,000 or higher

1. **Do you currently have health insurance?**

- Yes I have private insurance (for example, through my job, HMO)
- Yes I have public insurance (for example, Medicaid, Medicare)
- No

1. **Do you have a doctor, nurse, or other practitioner who you think of as your primary care provider?**

- Yes
- No
- Not sure

1. **How many biological children do you have?**

____________________________

1. **Have you previously received a cancer diagnosis before?**

- Yes
- No

1. **Has someone in your family been diagnosed with cancer before?**

- Yes
- No
- Don’t Know

1. **If you answered Yes to Question 14 or 15, what type(s) of cancer(s) were diagnosed?**

_________________________________________

1. **Have you received genetic testing before (carrier screening or cancer predisposition testing) in a clinical setting?**

- Yes
- No

**17a. If so, what type of testing have you received?**

________________________________________

1. **Has your primary care doctor ever discussed with you genetic testing?**
   - Yes
   - No
   - Don’t Know
2. **Have you received genetic testing before from a direct-to-consumer company (23&me, Ancestry.com, Color, etc)?**

- Yes
- No

1. **Has someone in your family (parents, siblings, aunts/uncles, cousins, children) undergone genetic testing and found a genetic mutation (like BRCA1/2)?**

- Yes
- No
- Don’t Know

**Carrier Screening**

Carrier screening is a type of genetic test that can tell you whether you carry a gene for certain genetic disorders. It generally is helpful for planned pregnancies of couples to determine the risk for a genetic disorder for a potential child. However, it can also be done at any time based upon family history in a clinical setting, or more recently from Direct-to-Consumer companies like Color, 23&me, and Ancestry. In a clinical setting however, generally results are returned by genetic counselors with guidance (if needed).

Below, we have some questions we’d like you to answer relating to carrier screening. Please select the box to the corresponding scale for your response.

| *Question* | **Unpleasant** | **Somewhat unpleasant** | **Neutral** | **Somewhat Pleasant** | **Pleasant** |
| --- | --- | --- | --- | --- | --- |
| Do you see carrier screening as |  |  |  |  |  |
|  | BREAK FOR NEW SCALE | | | | |
|  | **Unlikely** | **Somewhat Unlikely** | **Unsure** | **Somewhat Likely** | **Likely** |
| My use of carrier screening will be unpleasant |  |  |  |  |  |
| My use of carrier screening will produce scary results |  |  |  |  |  |
| Even with uncertainty of results I would still undergo carrier screening |  |  |  |  |  |
|  | BREAK FOR NEW SCALE | | | | |
|  | **Bad** | **Somewhat Bad** | **Neither Bad nor Good** | **Somewhat Good** | **Good** |
| Carrier screening is |  |  |  |  |  |
| If I used carrier screening it would be |  |  |  |  |  |
|  | BREAK FOR NEW SCALE | | | | |
|  | **Disagree** | **Somewhat Disagree** | **Neutral** | **Somewhat Agree** | **Agree** |
| Most people approve using carrier screening |  |  |  |  |  |
| My own family and friends would approve using carrier screening |  |  |  |  |  |
| Generally, I do what is what my family and/or friends think I should do |  |  |  |  |  |
| Most people use carrier screening |  |  |  |  |  |
| My own family and friends use carrier screening |  |  |  |  |  |
| My primary care doctor (or other healthcare providers) approve using carrier screening |  |  |  |  |  |
| My primary care doctor (or other healthcare providers) would approve ***me*** using carrier screening |  |  |  |  |  |
| Generally, I do what my what primary care doctor (or healthcare providers) thinks I should do |  |  |  |  |  |
|  | BREAK FOR NEW SCALE | | | | |
|  | **Difficult** | **Somewhat Difficult** | **Neither Difficult nor Easy** | **Somewhat Easy** | **Easy** |
| Getting carrier screening would be |  |  |  |  |  |
| Uncertainty of results would make carrier screening |  |  |  |  |  |
|  | BREAK FOR NEW SCALE | | | | |
|  | **Not Confident at all** | **Slightly Confident** | **Somewhat confident** | **Fairly confident** | **Completely Confident** |
| I could use carrier screening if I wanted |  |  |  |  |  |
| Even with challenges to testing I still could get carrier screening |  |  |  |  |  |
|  | BREAK FOR NEW SCALE | | | | |
|  | **Unlikely** | **Somewhat Unlikely** | **Unsure** | **Somewhat Likely** | **Likely** |
| In the next year, do you intent to use carrier screening in a clinical setting? |  |  |  |  |  |

**Cancer Predisposition Testing**

Cancer Predisposing Testing is a type of genetic testing for cancer risk that is typically ordered by a doctor (or genetic counselors in some states) involving testing for inherited genetic variants (genes) that are associated with a high to moderate increased risk of cancer. These tests generally are ordered in clinical settings (for more rigorous testing processes), with results interpreted and communicated through genetic counselors.

Below, we have some questions we’d like you to answer relating to carrier screening. Please select the box to the corresponding scale for your response.

| *Question* | **Unpleasant** | **Somewhat unpleasant** | **Neutral** | **Somewhat Pleasant** | **Pleasant** |
| --- | --- | --- | --- | --- | --- |
| Do you see cancer predisposition testing as |  |  |  |  |  |
|  | BREAK FOR NEW SCALE | | | | |
|  | **Unlikely** | **Somewhat Unlikely** | **Unsure** | **Somewhat Likely** | **Likely** |
| My use of cancer predisposition testing will be unpleasant |  |  |  |  |  |
| My use of cancer predisposition testing will produce scary results |  |  |  |  |  |
| Even with uncertainty of results I would still undergo cancer predisposition testing |  |  |  |  |  |
|  | BREAK FOR NEW SCALE | | | | |
|  | **Bad** | **Somewhat Bad** | **Neither Bad nor Good** | **Somewhat Good** | **Good** |
| Cancer predisposition testing is |  |  |  |  |  |
| If I used cancer predisposition testing it would be |  |  |  |  |  |
|  | BREAK FOR NEW SCALE | | | | |
|  | **Disagree** | **Somewhat Disagree** | **Neutral** | **Somewhat Agree** | **Agree** |
| Most people approve using cancer predisposition testing |  |  |  |  |  |
| My own family and friends would approve using cancer predisposition testing |  |  |  |  |  |
| Generally, I do what is what my family and/or friends think I should do |  |  |  |  |  |
| Most people use cancer predisposition testing |  |  |  |  |  |
| My own family and friends use cancer predisposition testing |  |  |  |  |  |
| My primary care doctor (or other healthcare providers) approve using cancer predisposition testing |  |  |  |  |  |
| My primary care doctor (or other healthcare providers) approve ***me*** using cancer predisposition testing |  |  |  |  |  |
| Generally, I do what my what primary care doctor (or healthcare providers) thinks I should do |  |  |  |  |  |
|  | BREAK FOR NEW SCALE | | | | |
|  | **Difficult** | **Somewhat Difficult** | **Neither Difficult nor Easy** | **Somewhat Easy** | **Easy** |
| Getting cancer predisposition testing would be |  |  |  |  |  |
| Uncertainty of results would make cancer predisposition testing |  |  |  |  |  |
|  | BREAK FOR NEW SCALE | | | | |
|  | **Not Confident at all** | **Slightly Confident** | **Somewhat confident** | **Fairly confident** | **Completely Confident** |
| I could use cancer predisposition testing if I wanted |  |  |  |  |  |
| Even with challenges to testing I still could get cancer predisposition testing |  |  |  |  |  |
|  | BREAK FOR NEW SCALE | | | | |
|  | **Unlikely** | **Somewhat Unlikely** | **Unsure** | **Somewhat Likely** | **Likely** |
| In the next year, do you intent to use cancer predisposition testing in a clinical setting? |  |  |  |  |  |
